# Prevalence of liver fibrosis and cirrhosis in 699 Moroccan patients with chronic hepatitis C

**DOI:** 10.11604/pamj.2021.39.32.21235

**Published:** 2021-05-12

**Authors:** Abdellatif Bouayad, Fatima Zahra Laamiri, Lahcen Elmoumou, Bouchra Rezzouk, Rachid Hadef

**Affiliations:** 1 Faculty of Medicine and Pharmacy of Oujda, Mohamed First University, Oujda, Morocco,; 2 Laboratory of Immunology, Pasteur Institute of Morocco, Casablanca, Morocco,; 3 Hassan First University, Higher Institute of Health Sciences of Settat, Health Sciences and Technology Laboratory, Settat, Morocco,; 4 Higher Institute of Nursing Professions and Technical Health, Tiznit, Morocco,; 5 Laboratory of Microbiology and Molecular Biology, Faculty of Sciences, Mohammed V University, Rabat, Morocco,; 6 Laboratory of Hematology, Mohamed V military Hospital, Faculty of Medicine and Pharmacy, Rabat, Morocco

**Keywords:** Chronic hepatitis C, cirrhosis, fibrosis, fibro test, Moroccan patients

## Abstract

**Introduction:**

chronic hepatitis C (CHC) can cause severe complications, including fibrosis and cirrhosis. Very little is known about the prevalence of these complications in the Moroccan population.

**Methods:**

the prevalence of liver fibrosis and cirrhosis using a non-invasive blood test (FibroTest and ActiTest) was studied in 699 Moroccan patients with CHC for 4 years (from January 2014 to December 2017). The serum immunological markers: α2-macroglobulin, haptoglobin, apolipoprotein A1 were analyzed nephelometrically on BN ProSpec® System. The serum biochemical markers: γ-glutamyltransferase, alanine aminotransferase, and bilirubin were performed using the VITROS® Chemistry System Ortho Clinical Diagnostic. A 699 patients with CHC were identified.

**Results:**

the overall prevalence of cirrhosis (F4) was estimated at 31.8%. Thirteen point nine percent (13.9%) of patients with cirrhosis had a risk of developing esophageal varices and a 3.3% risk of developing primary liver cancer. The association between cirrhosis and age showed an increase in prevalence after age 55 years old [OR=7.68(95%CI=4.9-12.2); p<0.0001]. No significant association for cirrhosis was found for sex.

**Conclusion:**

according to the results of FibroTest, 32% of patients with CHC had cirrhosis. The older age was independently associated with liver cirrhosis.

## Introduction

Chronic hepatitis C (CHC) is a public health chronic disease, affecting an estimated 0.2% to 2% of the Moroccan general population [1]. It predisposes these individuals to several complications such as fibrosis, cirrhosis, or even death. Rapidly progressive liver fibrosis is an early indicator of CHC severity [2]. According to histological METAVIR scoring, the estimate of fibrosis progresses use principally transition rates between successive stages from the healthy liver (F0) to cirrhosis (F4) [2,3]. The last stage includes subjects with a broad spectrum of severity.

Diagnosis of fibrosis facilitates the early therapeutic intervention that prevents irreversible liver damage for patients with CHC. Although liver biopsy is considered the gold standard for evaluating fibrosis and cirrhosis stages, it is an invasive procedure and leads to complications in 0.6-5.0% of patients [4,5]. Thus, it is not an adequate intervention for monitoring liver damage for patients with CHC. The new non-invasive biomarkers using immunological and biochemical markers showed high positive predictive values for significant illness in patients with CHC [6]. The FibroTest/ActiTest is the most noninvasive test used for staging severity of liver fibrosis and cirrhosis according to the METAVIR scoring system. FibroTest allows estimate liver fibrosis and ActiTest - necroinflammatory activity. Moreover, it is also validated as a quantitative parameter in predicting prognosis for the occurrence of cirrhosis and their major complications [7]. Since CHC and its complications are considered a major public health problem in Morocco and few studies have been carried to assess the prevalence of liver fibrosis and cirrhosis; this cross-sectional study was aimed to analyze the prevalence of fibrosis and cirrhosis in 699 Moroccan patients with CHC.

## Methods

In this cross-sectional study, 699 Moroccan patients with CHC were selected randomly from the Pasteur Institute for 4 years between January 2014 and December 2017. We included patients with anti HCV antibodies and HCV RNA positive. Patients with confounding variables such as hepatotoxic medical drugs, co-infection with other infectious diseases (e.g. hepatitis B virus, human immunodeficiency virus-1/2) as well as auto-immune hepatitis and primary biliary cirrhosis were excluded in this study. The study protocol was approved by an institutional/local ethics committee at the Pasteur Institute of Morocco and funded by the Moroccan Ministry of Health for epidemiological purposes. Patients´ informed consent was signed before the inclusion of patients in the study.

The serum immunological markers: α2-macroglobulin, haptoglobin, apolipoprotein A1 were analyzed nephelometrically on BN ProSpec® System. The serum biochemical markers: γ-glutamyltransferase, alanine aminotransferase, and total bilirubin were performed using the VITROS® Chemistry System Ortho Clinical Diagnostic. Patients´ data like age and gender were required to generate the fibrosis and inflammation stages in the liver. FibroTest/ActiTest scores were computed on the Biopredictive website and the results were provided with security algorithms. The result of the different markers is expressed in a score varying from 0 to 1 in proportion to fibrosis severity and inflammation activity with a conversion to METAVIR stage according to the following scheme: for FibroTest®, for non-cirrhotic stages; F0 (0 to ≤0.28) to no fibrosis, F1 (>0.28 to ≤0.48) to minim fibrosis, F2 (>0.8 to ≤0.58) to moderate fibrosis, F3 (>0.58 to ≤0.74) to advanced fibrosis [8]; and for the new cirrhotic stages, F4.1 (>0.74 to ≤0.85) to uncomplicated cirrhosis, F4.2 (>0.85 to ≤0.95) to cirrhosis with varices and without severe events and F4.3 (>0.95-1.00) to cirrhosis with severe events [9]. For ActiTest®, A0 to no activity, A1 to minim activity, A2 to important activity, and A3 to severe activity. According to criteria given by BioPredictive, significant hepatic activity is defined as A ≥2 (score > 0.52).

The data was performed by SPSS for Windows version 16.0 (SPSS, Chicago, IL, USA). Values are expressed mean±SD or number (percentage). We compared the demographic and disease characteristics relating to the criteria given by BioPredictive using Student´s t-test and Chi-Deux -test as appropriate. Odds ratios (OR) and their 95% confidence intervals (CI) were also calculated. All potential predictors of fibrosis and cirrhosis with P < 0.3 were assessed using the logistic regression diagnostics procedure. Uncorrected p-values below 0.05 were considered as statistically significant.

## Results

**Study population:** the descriptive features of the patients are shown in Table 1. A total of 699 patients with CHC were included; 62.5% of whom were women. The mean age of patients was 59 years (range 51-68). Among presumed non-cirrhotic patients, the following distribution of FibroTest scores was observed: F0 (no fibrosis or minim fibrosis) in 145 patients (20.7%), F1 in 133 patients (19%), F2 (moderate fibrosis) in 75 patients (10.7%), and F3 (advanced fibrosis) in 124 patients (17.7%). The overall prevalence of cirrhosis (F4) was estimated at 31.8%. Among presumed cirrhotic patients, cirrhosis without varices or severe events (F4.1) occurred in 102 patients (14.6%), cirrhosis with varices and without severe events (F4.2) in 97 patients (13.9%), and cirrhosis with severe event (F4.3) in 23 patients (3.3%). The distribution of ActiTest scores of A0-A1 was 49.2%, A1-A2 was 16.2% and A2-A3 was 34.7%.

**Table 1:** demographic and medical characteristics of patients with chronic hepatitis C

Characteristics	Patients (n=699)
Age in years (range)^a^	59 (51-68)
**Gender^b^**	
Male	262 (37.5)
Female	437 (62.5)
**Stage of fibrosis^b^**	
No fibrosis (F0)	145 (20.7)
Minim fibrosis (F1)	133 (19.0)
Moderate fibrosis (F2)	75 (10.7)
Advanced fibrosis (F3)	124 (17.7)
Cirrhosis (F4)	222 (31.8)
**Stage of cirrhosis^b^**	
No complication (F4.1)	102 (14.6)
Varices (F4.2)	97 (13.9)
Cirrhosis with severe event (F4.3)	23 (3.3)
**Necroinflammatory activity^a^**	
Insignifiant activity (A < 2)	290 (41.5)
Signifiant activity (A ≥ 2)	409 (58.5)

Note: ^a^Values are expressed as median and interquartile, ^b^Values are expressed as count and percentage

**Univariate analysis:** the results of univariate logistic regression analysis of factors influencing cirrhosis are shown in Table 2. No significant association for cirrhosis was found for sex. In contrast, age at diagnostic [OR=1.07 (95%CI=1.05-1.08); p<0.0001] was significantly associated with cirrhosis. Moreover, patients aged over 55 years tended to have cirrhosis more frequently compared with younger patients [89.2% vs 51.8%; OR=7.68(95%CI=4.9-12.2); p<0.0001]. Finally, patients with significant necroinflammatory activity were much more likely to develop cirrhosis [OR=24.4(95%CI=13.3-45); p<0.0001].

**Table 2:** univariate analysis of factors influencing cirrhosis

Demographic characteristics	No cirrhosis (n= 477)	Cirrhosis (n=222)	OR*	95% CI	p-value
Median age (years)^a^	56 (45-65)	66 (59.7-72)	1.07	1.05-1.08	< 0.0001
**Age group (years)^b^**					
<55	230 (48.2)	24 (19.8)	7.68	4.9-12.2	< 0.0001
≥55	247 (51.8)	198 (89.2)			
**Gender^b^**					
Male	304 (63.7)	133 (59.9)	1.2	0.8-1.6	0.33
Female	173 (36.3)	89 (40.1)			
**Necroinflammatory activity^b^**					
Insignificant activity	278 (58.3)	12 (5.4)	24.4	13.3-45	< 0.0001
Significant activity	199 (41.7)	210 (94.6)			

Note: ^a^Values are expressed as median and interquartile; ^b^Values are expressed as count and percentage;* Odds ratio (OR) calculated using logistic regression; Significance threshold p<0.05; CI: confidence interval

## Discussion

This is the first study to estimate the frequency of fibrosis and cirrhosis in Moroccan patients with CHC according to FibroTest and ActiTest. Staging the severity of liver fibrosis according to the METAVIR scoring system is essential to define the prognosis and management of CHC disease. In our study, 17.7% of Moroccan adults affected with CHC have diagnosed advanced fibrosis and 31.8% have liver cirrhosis. Worldwide, cirrhosis is the major risk of mortality, accounting for approximately 800,000 deaths annually [10]. This prevalence remains relatively higher when compared to epidemiological studies in the literature. It has been previously estimated that advanced fibrosis and cirrhosis appear in alcohol-dependent patients with a respective percentage of 15.3% and 8.8% [11]. A general population study conducted by Fleming *et al*. [12] reported the prevalence of cirrhosis in the United Kingdom to be 68%, whereas a study by Scaglione *et al*. [13] estimated the prevalence at 0.27% in the United States.

Interestingly, our estimate was also excessively higher compared to what has been reported by Cacoub *et al*. [14] in which the estimated cirrhosis rate was 2% by liver biopsy. This apparently discordant result in the epidemiology of cirrhosis may be explained by distinctness in statistical methods applied and the higher prevalence of HCV in Moroccan patients (7.7%) [14]. In addition, the current limitations of FibroTest/ActiTest are their inability to distinguish between intermediate stages of fibrosis. Overall, however, the performances of FibroTest and ActiTest are excellent as compared to liver biopsy for the management of advanced fibrosis or cirrhosis [15] and providing critical information to clinicians without exposing patients to the adverse effects of liver biopsy. In CHC, FT provides validated non-invasive and quantitative markers of predicting the presence of cirrhosis complications like esophageal varices, primary liver cancer, variceal bleeding, and the “hepatic insufficiency” complications (ascites, jaundice, encephalopathy) [7]. The two complications mainly requiring screening for a patient with established liver cirrhosis are primary liver cancer and esophageal varices. In our study, we observed that patients with cirrhosis had a 13.9% risk of developing esophageal varices and a 3.3% risk of developing primary liver cancer. Thierry Poynard *et al*. suggest that patients with FT remaining <0.74 (F4.1) do not require esophagogastroduodenoscopy and patients with FT >0.95 had a 14% risk of developing varices at 5 years [16].

It was reported that cirrhosis rates peaking during the fourth and the fifth decade and then again after the age of 75. As expected, hepatitis C, alcohol, and diabetes play a large role in the epidemiology of cirrhosis, accounting for 53.5% of cases [13]. In our study, the results of the association between cirrhosis and age showed an increase in prevalence after 55 years old. This higher prevalence rate of cirrhosis in older people could be attributed to longer exposure to risk factors for HCV transmission, higher rates of poverty, lower school attainment, and a delay in diagnostic and therapeutic intervention. This is in agreement with a study performed by Pineda *et al*. who shows that advanced liver fibrosis was more prevalent in old age [15]. A higher fibrosis rate, which happens in the liver, could be attributed to the loss of regenerative and homeostatic capacity of the liver over the age. Notably, senescent hepatocytes have been involved in fibrosis progression and cirrhosis [17,18].

Another important finding in our study is that cirrhotic patients are at increased risk for developing a systemic inflammatory response with significant necro-inflammatory activity. It is speculated that during cirrhosis, damage-associated molecular patterns (DAMPs) and cell contents released from necrotic hepatocytes through necroptosis as well as the persistence of immune cell activation, may overstimulate the innate immune response and lead to systemic inflammation [19,20]. Moreover, acute-phase protein serum levels including α2-macroglobulin, haptoglobin, apolipoprotein A1 have been increased during the inflammatory reaction, and their components contribute to innate immune reaction against infectious diseases.

No significant difference in cirrhosis according to gender was found in our study. Based on the data generated in our study and determination of factors independently associated with cirrhosis and to face this growing epidemic, we can assume that the establishment of screening programs and interventions in the general population and particularly in a subgroup with CHC will allow for wider prevention of irreversible liver damage. The limitations of our current study include: first, in our context with higher rates of poverty, unable to perform α-fetoprotein (AFP) and liver ultrasonography for screening hepatocellular carcinoma in all subjects with cirrhosis, and second, FT performance was not compared to the hepatic venous pressure gradient, which is the best prognostic indicator of the formation of varices. However, these results provide information that may be used for further analysis of cirrhosis studies in Moroccan patients.

## Conclusion

The results of our study suggested that the rates of liver cirrhosis was higher in Moroccan CHC patients. In this group, the advanced age was a risk factor for developing cirrhosis disease. Our observation suggest also that other test with higher sensitivity need to be implemented for the detection and monitoring of liver cirrhosis.

### What is known about this topic

- Until now, the global assessment for the cirrhosis and fibrosis in patients with chronic infection with the hepatitis C virus, using liver biopsy is difficult to perform in daily clinical practice in Moroccan context.

### What this study adds

- We propose that new noninvasive biomarkers, including use of fibrosis biomarker (FibroTest™), may allow reliable and efficient evaluation of the fibrosis stages and cirrhosis complications in Moroccan patients.

## References

[1] Fadlalla FA, Mohamoud YA, Mumtaz GR, Abu-Raddad LJ (2015). The epidemiology of hepatitis C virus in the Maghreb region: systematic review and meta-analyses. PloS one.

[2] Poynard T, Bedossa P, Opolon P (1997). Natural history of liver fibrosis progression in patients with chronic hepatitis C. The OBSVIRC METAVIR CLINIVIR, and DOSVIRC groups. Lancet.

[3] Thein HH, Yi Q, Dore GJ, Krahn MD (2008). Estimation of stage-specific fibrosis progression rates in chronic hepatitis C virus infection: a meta-analysis and meta-regression. Hepatology.

[4] Gunneson TJ, Menon KV, Wiesner RH, Daniels JA, Hay JE, Charlton MR (2002). Ultrasound-assisted percutaneous liver biopsy performed by a physician assistant. Am J Gastroenterol.

[5] Cakmakci E, Caliskan KC, Tabakci ON, Tahtabasi M, Karpat Z (2013). Percutaneous liver biopsies guided with ultrasonography: a case series. Iran J Radiol.

[6] Poynard T, Morra R, Ingiliz P, Imbert-Bismut F, Thabut D, Messous D (2008). Assessment of liver fibrosis: noninvasive means. Saudi J Gastroenterol.

[7] Ngo Y, Munteanu M, Messous D, Charlotte F, Imbert-Bismut F, Thabut D (2006). A prospective analysis of the prognostic value of biomarkers (FibroTest) in patients with chronic hepatitis C. Clin Chem.

[8] Poynard T, Morra R, Halfon P, Castera L, Ratziu V, Imbert-Bismut F (2007). Meta-analyses of FibroTest diagnostic value in chronic liver disease. BMC Gastroenterol.

[9] Garcia-Tsao G, Friedman S, Iredale J, Pinzani M (2010). Now there are many (stages) where before there was one: in search of a pathophysiological classification of cirrhosis. Hepatology.

[10] Bosetti C, Levi F, Lucchini F, Zatonski WA, Negri E, La Vecchia C (2007). Worldwide mortality from cirrhosis: an update to 2002. J Hepatol.

[11] Gudowska M, Wojtowicz E, Cylwik B, Gruszewska E, Chrostek L (2015). The distribution of liver steatosis, fibrosis, steatohepatitis and inflammation activity in alcoholics according to FibroMax Test. Adv Clin Exp Med.

[12] Fleming KM, Aithal GP, Solaymani-Dodaran M, Card TR, West J (2008). Incidence and prevalence of cirrhosis in the United Kingdom, 1992-2001: a general population-based study. J Hepatol.

[13] Scaglione S, Kliethermes S, Cao G, Shoham D, Durazo R, Luke A (2015). The epidemiology of cirrhosis in the United States: a population-based study. J Clin Gastroenterol.

[14] Cacoub P, Ohayon V, Sekkat S, Dumont B, Sbai A, Lunel F (2000). Epidemiologic and virologic study of hepatitis C virus infections in Morocco. Gastroenterol Clin Biol.

[15] de Lédinghen V, Poynard T, Wartelle C, Rosenthal E (2008). Non-invasive evaluation of liver fibrosis in hepatitis C. Gastroenterol Clin Biol.

[16] Poynard T, Vergniol J, Ngo Y, Foucher J, Munteanu M, Merrouche W (2014). Staging chronic hepatitis C in seven categories using fibrosis biomarker (FibroTest™) and transient elastography (FibroScan®). J Hepatol.

[17] Huda N, Liu G, Hong H, Yan S, Khambu B, Yin XM (2019). Hepatic senescence, the good and the bad. World J Gastroenterol.

[18] Aravinthan AD, Alexander GJM (2016). Senescence in chronic liver disease: is the future in aging?. J Hepatol.

[19] Dirchwolf M, Ruf AE (2015). Role of systemic inflammation in cirrhosis: from pathogenesis to prognosis. World J Hepatol.

[20] Laleman W, Claria J, Van der Merwe S, Moreau R, Trebicka J (2018). Systemic inflammation and acute-on-chronic liver failure: too much, not enough. Can J Gastroenterol Hepatol.

